# Refining the ITBCC tumor budding scoring system with a “zero-budding” category in colorectal cancer

**DOI:** 10.1007/s00428-021-03090-w

**Published:** 2021-04-12

**Authors:** Inti Zlobec, Melanie Bächli, Francesca Galuppini, Martin D. Berger, Heather E. Dawson, Iris D. Nagtegaal, Alessandro Lugli

**Affiliations:** 1 Institute of Pathology, University of Bern, Murtenstrasse 31, 3008 Bern, Switzerland; 2 Pathology Unit, Department of Medicine (DIMED), University of Padua, Via Gabelli, 61 35121 Padova, Italy; 3 Department of Medical Oncology, Inselspital, Bern University Hospital, University of Bern, Freiburgstrasse 41G, Bern, 3010 Switzerland; 4 Radboud Institute for Molecular Life Sciences, Radboudumc, Department of Pathology, 812 Geert Grooteplein, Nijmegen, 6500 HB Netherlands

**Keywords:** Tumor budding, Colorectal cancer

## Abstract

Tumor budding scoring guidelines from the International Tumor Budding Consensus Conference (ITBCC) for colorectal cancer propose three groups: BD1 (0–4 buds/0.785 mm^2^), BD2 (5–9 buds/0.785 mm^2^), and BD3 (10 or more buds/0.785 mm^2^). Here, we investigate whether a fourth scoring category, namely zero buds, may have additional clinical relevance. The number of tumor buds/0.785 mm^2^ was scored in 959 cases. Those with zero tumor buds were considered BD0, while a new BD1 category of 1–4 buds was proposed. Associations of both scoring approaches with clinicopathological features were analyzed. Conventional ITBCC scoring showed expected associations with unfavorable histopathological prognostic factors. In total, 111/959 (11.6%) were BD0. A significant difference was found when BD0 was compared statistically to BD1 (1–4 buds) for pT, TNM, tumor grade, and lymphatic, venous, and perineural invasion (*p* < 0.01, all). Tumors with BD0 occur relatively frequently and contribute additional information on tumor behavior. BD0 should be considered for subsequent ITBCC guidelines.

## Introduction

Tumor budding is an important prognostic factor in the clinical management of cancer patients [1]. Although best described in colorectal cancers, evidence indicates that patients with a variety of different tumor types suffer from more aggressive disease and worse overall and disease-free survival in the context of high-grade tumor budding [1]. The definition of “high-grade” may vary depending on the clinical endpoint of interest. The International Tumor Budding Consensus Conference (ITBCC) guidelines describe three different budding grades, termed BD1, BD2, and BD3, which consist of 0–4, 5–9, and 10 or more buds in a hotspot of 0.785 mm^2^, respectively [2]. For pT1 colorectal cancers, the clinically relevant threshold for identifying patients who may benefit from a surgical rather than endoscopic resection is low-risk BD1 versus high-risk BD2 + BD3. In contrast, to identify patients with high-risk stage II colorectal cancers, the threshold is greater, namely low-risk (BD1 + BD2) versus high-risk BD3 [2]. Still an on-going matter of debate is the necessity for a fourth BD category in colorectal cancers: BD0. This category would highlight tumors with complete absence of tumor budding all together. Not only could BD0 underline an important clinical subgroup but also would shed light on the biology of the tumor budding phenotype in general.

The aim of this study was to determine the frequency and potential clinical relevance of a BD0 (zero peritumoral buds) category for primary surgically treated colorectal cancer patients, with the goal to further refine the current ITBCC grading scheme for tumor budding.

## Methods

### Materials and methods

**Fig. 1:**
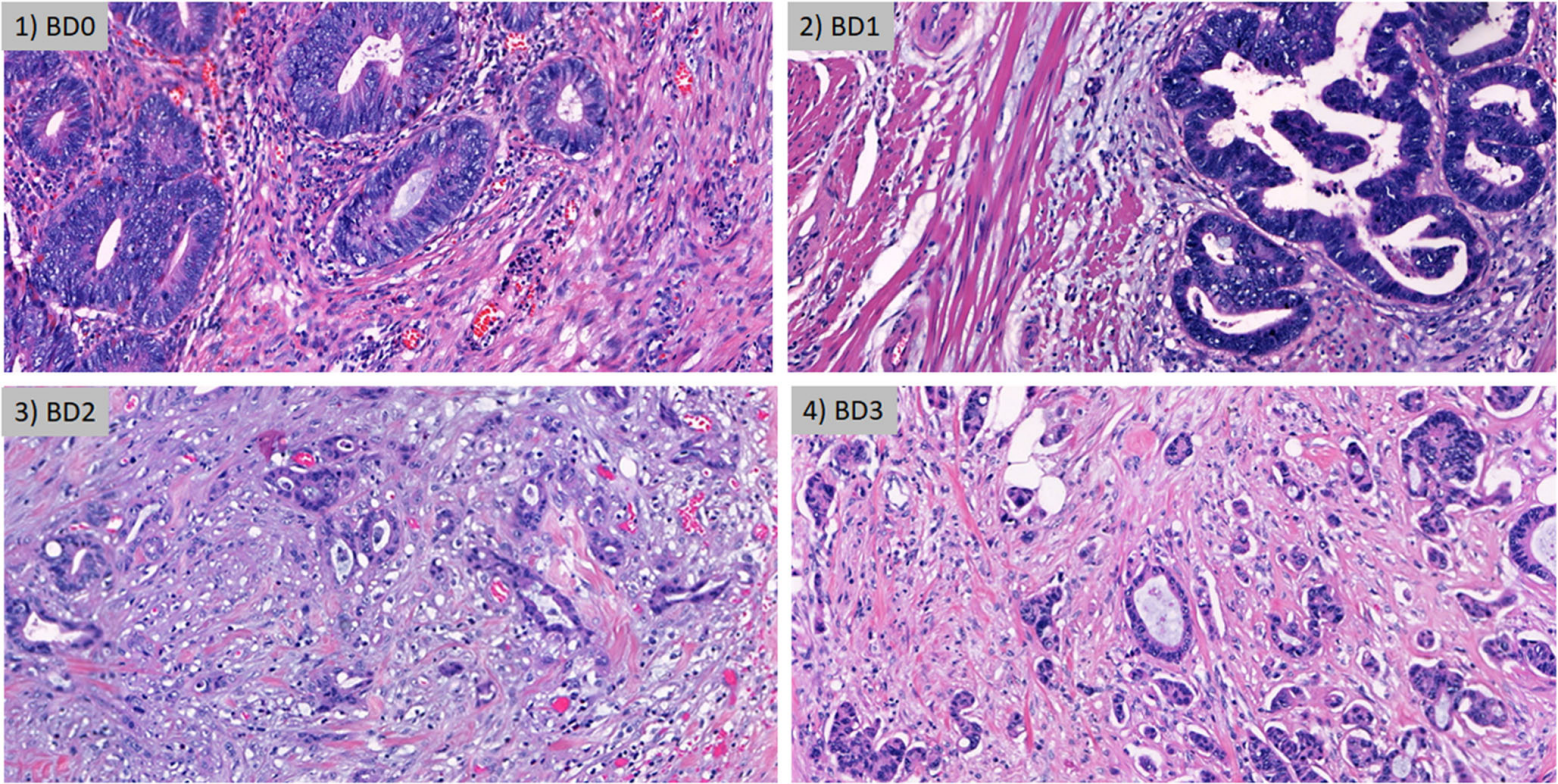
Representative H&E images of 1) BD0, 2) BD1, 3) BD2, and 4) BD3 tumor budding scores in colorectal cancer (×20 objective magnification)

All patients with primary colorectal cancer diagnosed at the Institute of Pathology, University of Bern, from 2002 to April 2019 were originally considered. Of those 1873 consecutive patients, those receiving pre-operative chemoradiotherapy as well as those with missing tumor budding scores were excluded from further study, leaving 959 patients. For patients from 2002 to 2016 (*n* = 261), all H&E slides of the corresponding resection specimens were re-reviewed by expert pathologists, blinded to the clinical outcome. Histopathological information for each tumor from 2016 to 2019 (*n* = 698) was obtained from diagnostic records, reported by different attending pathologists. This information includes patient gender, the histological subtype of the tumor, the tumor grade, pT, pN, venous (V), lymphatic (L), perineural invasion (PNI), and the presence of metastasis (clinical or pathological) at the time of first diagnosis. The Klintrup-Mäkinen score is also given, which indicates the degree of peritumoral inflammation on H&E slides and is shown to be a reproducible and stage-independent prognostic factor [3]. As per the ITBCC criteria, each case was assigned a budding count, referring to the total number of buds identified in the densest 0.785 mm^2^ area and subsequently categorized into BD1 (0–4 buds), BD2 (5–9 buds), and BD3 (10 or more buds) [2]. In order to determine the added value of a zero-budding category, we performed a second categorization. Here, we further classify cases with zero tumor buds (BD0) followed by tumors with 1–4 tumor buds, denoted as BD1* in this manuscript. BD2 and BD3 remain the same. Representative images of BD categories can be found in Fig. 1. Only patients with ITBCC scores were included in the final analysis, leaving 959 patients. All data are summarized in Table 1.

**Table 1:** Patient characteristics and association of budding score with clinicopathological data (*n* = 959). BD0 = zero tumor buds, BD1* indicates 1–4 tumor buds, BD2 represent 5–9 buds, and BD3 are 10 buds or more

				BD				
Feature		Total	BD0 (*n* = 111; 11.6%)	BD1* (*n* = 358; 37.3%)	BD2 (*n* = 224 23.3%)	BD3 (*n* = 266; 27.7%)	*p* value	*p* value for difference BD0 and BD1*
Gender	Male	556 (58.0)	59 (53.2)	202 (56.4)	140 (62.5)	155 (58.5)	0.3470	0.5444
	Female	403 (42.0)	52 (46.9)	156 (43.6)	84 (37.5)	110 (41.6)		
Histological subtype	Adenocarcinoma	855 (89.3)	96 (87.3)	311 (87.1)	205 (91.5)	242 (91.3)	0.0659	0.438
	Mucinous	87 (9.1)	14 (12.7)	41 (11.5)	16 (7.1)	16 (6.0)		
	Other	15 (1.6)	0 (0.0)	5 (1.4)	3 (1.3)	7 (2.6)		
Tumor location	Left	423 (45.9)	49 (48.5)	157 (44.9)	105 (49.1)	111 (43.2)	0.6663	0.483
	Rectum	142 (15.4)	14 (13.9)	54 (15.4)	34 (15.9)	40 (15.6)		
	Right	357 (51.3)	36 (35.6)	136 (38.9)	74 (34.6)	105 (40.9)		
pT	pT1	139 (14.6)	50 (46.3)	59 (16.6)	15 (6.8)	15 (5.7)	<0.0001	<0.0001
	pT2	132 (13.9)	21 (19.4)	65 (18.3)	22 (9.9)	24 (9.1)		
	pT3	440 (46.2)	30 (27.8)	177 (49.7)	126 (56.8)	107 (40.4)		
	pT4	241 (25.3)	7 (6.5)	55 (15.5)	59 (26.6)	119 (44.9)		
pN	pN0	497 (55.7)	75 (84.3)	239 (72.2)	107 (50.5)	75 (29.0)	<0.0001	0.065
	pN1 + 2	395 (44.3)	14 (15.7)	91 (21.8)	105 (49.5)	184 (71.0)		
pM or cM	Absent	274 (77.4)	32 (91.4)	100 (90.1)	74 (81.3)	68 (58.6)	<0.0001	0.815
	Present	80 (22.6)	3 (8.6)	11 (9.9)	17 (18.7)	48 (41.4)		
TNM stage	I	112 (18.3)	25 (48.1)	51 (22.9)	21 (14.2)	15 (8.0)	<0.0001	0.0026
	II	201 (32.9)	14 (26.9)	102 (45.7)	52 (35.1)	33 (17.7)		
	III	206 (33.7)	10 (19.2)	58 (26.0)	58 (39.2)	80 (42.8)		
	IV	92 (15.1)	3 (5.8)	12 (5.4)	17 (11.5)	59 (31.6)		
Tumor grade	G1	106 (11.3)	37 (34.6)	45 (13.0)	18 (8.2)	6 (2.3)	<0.0001	<0.0001
	G2	656 (70.1)	50 (46.7)	247 (71.2)	169 (76.8)	189 (72.4)		
	G3	174 (18.6)	20 (18.7)	55 (15.9)	33 (15.0)	66 (25.3)		
Lymphatic	L0	452 (47.3)	91 (82.7)	234 (65.6)	81 (36.5)	46 (17.4)	<0.0001	0.0006
	L1	503 (52.7)	19 (17.3)	123 (34.5)	141 (63.5)	219 (82.6)		
Venous	V0	533 (55.8)	97 (87.4)	241 (67.5)	106 (47.8)	89 (33.6)	<0.0001	<0.0001
	V1	423 (44.3)	14 (12.6)	116 (32.5)	116 (52.3)	176 (66.4)		
Perineural	Pn0	769 (81.0)	109 (99.1)	325 (91.8)	177 (80.1)	157 (59.5)	<0.0001	0.0067
	Pn1	181 (19.1)	1 (0.9)	29 (8.2)	44 (19.9)	107 (40.5)		
MMR	Deficient	100 (15.5)	12 (17.7)	43 (18.1)	19 (12.2)	26 (14.3)	0.3847	0.9252
	Proficient	544 (84.5)	56 (82.4)	194 (81.9)	137 (87.8)	156 (85.7)		
Klintrup-Mäkinen								
	0	32 (3.3)	4 (3.6)	10 (2.8)	2 (0.9)	16 (6.0)		
	1	448 (46.7)	55 (49.6)	159 (44.4)	109 (48.7)	125 (47.0)	0.009	0.075
	2	330 (34.4)	39 (35.1)	119 (33.2)	74 (33.0)	98 (36.8)		
	3	149 (15.5)	13 (11.7)	70 (19.6)	39 (17.4)	27 (10.2)		

### Statistical analysis

We used a chi-square test to determine differences in budding categories in association with categorical data. Two analyses were performed each time, one analyzing differences in the distribution of features across BD1–BD3 (grouping BD0 together with BD1) and analyzing differences only between BD0 and BD1 to determine the additional value of this extended category. *p* values <0.05 were considered statistically significant. Kaplan-Meier curves and log-rank test were used to test for differences in survival times by BD category. Analyses were carried out using SAS (v9.4 The SAS Institute, Cary NC).

### Ethics

This study was approved by the ethics committee of the Canton of Bern KEK 2020–00498.

## Results

### Validation of ITBCC scores

Analysis of standard ITBCC BD1-BD3 indicated a very strong relationship between higher BD score and more advanced pT stage, lymph node metastasis, distant metastasis, higher tumor grade, lymphatic and venous vessel invasion, and perineural invasion (all *p* < 0.0001). Some differences between budding and Klintrup-Mäkinen score were identified, but did not show a consistent directionality. These results validate previous findings from the literature and support the appropriateness of the cohort.

### Added value of BD0

**Fig. 2:**
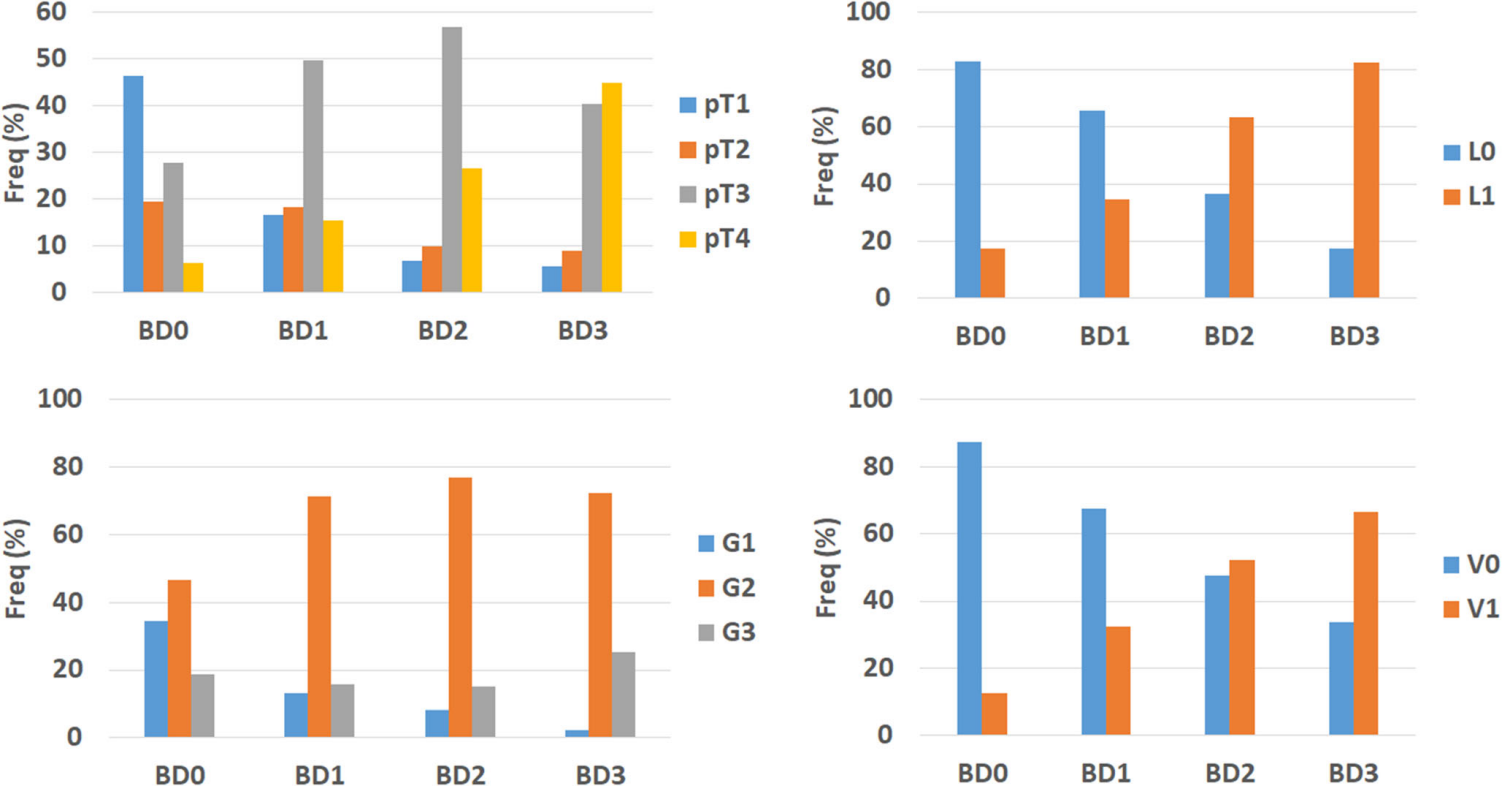
Distribution of BD0 category across (left upper) pT classification, (right upper) lymphatic vessel invasion, (bottom left) tumor grade, and (bottom right) venous vessel invasion

Of 959 patients, 111 (11.6%) were found to have BD0 cancers. The addition of BD category further strengthened the association between pT stage and tumor budding (Fig. 2), showing that 46.3% of pT1 cases were BD0, in contrast to 16.6%, 6.8%, and 5.7% of BD1, BD2, and BD3 cases (*p* < 0.0001). Furthermore, 34.6% of patients with BD0 tumors had low-grade tumors, in comparison to 13%, 8.2%, and 2.3% of BD1, BD2, and BD3 tumors (*p* < 0.0001). Similar results were found for lymphatic and venous invasion as well as perineural invasion (*p* < 0.01). Of the 139 pT1 cases, information (N0 or N1–2) on lymph node metastasis was present in 86 patients. Seventy-seven (89.5%) had no lymph node metastasis. Of the 9 remaining cases, 2 were BD0 in comparison to 7 with BD1*, BD2, and BD3 tumors. This difference was, however, not statistically significant (*p* = 0.4406).

### Overall (OS) and disease-free (DFS) survival information

OS and DFS were available for 361 and 351 patients, respectively, of which only 37 were BD0. Although tumor budding scores stratify patients into better or worse OS (*p* = 0.0035) and DFS (*p* = 0.0086), there was no significant difference between BD0 and BD1*.

## Discussion

The novel findings of this study show that >10% of all primarily surgically treated colorectal cancers have a complete absence of tumor budding and that these “BD0” cancers are far less aggressive than those with even a minimal number of tumor buds (1–4 tumor buds/0.785 mm^2^).

The phenomenon of tumor budding cannot be defined as a “categorical variable.” Some reports in adenocarcinomas (i.e., not signet ring cell histology) suggest the number of buds can range from zero to several hundred depending on the objective lens area [4]. Although it can be argued that a very large amount of tumor buds per defined area may be the result of cutting artifact, the absence of tumor buds along the entire border of the cancer can be explained by either [1] buds were overseen during diagnosis or [2] it is the result of a true pattern of tumor growth, or [3] in the case of pT1, tissues rather represented pseudoinvasion, which is excluded here, after careful re-examination of pT1 BD0. The reason for this growth pattern cannot solely be explained by the presence of peritumoral lymphocytic inflammation, which is described in our study by a high Klintrup-Mäkinen score, with values of 3 indicating thick bands of inflammatory infiltrates around the invasion edge of the cancer [3]. Previous works have shown that colorectal cancers with more “infiltrating” growth patterns are often high-grade budders and have little in terms of inflammation [5–7]. In contrast, those with pushing margins tend to have low budding and are much more heavily inflamed. In this study, we find that tumors with absence of budding (BD0) are independent of Klintrup-Mäkinen scores. Moreover, these tumors are not more likely to be MSI-H, which is characterized typically by a high number of intraepithelial T-lymphocytes. In fact, in contrast to previous works [8], the relationship between tumor budding and MSI-H here cannot be confirmed. This result is supported by work from other groups showing no difference in MSI status [9, 10]. Finally, although mucinous cancers have a more pushing tumor growth pattern, our findings again underline no relationship between budding and histological subtype. A previous work by our group has also shown that no changes in driver mutations can explain the presence of tumor budding [11]. Evidence from the tumor stroma points to epigenetic changes favoring the budding process, for example, methylation of TWIST1 and TWIST2 in stromal cells, or changes in miRNA has been found to occur in non-tumor budding regions. Evaluating the expression of MiR-21 in tumor buds, Moller and colleagues hypothesize that miR-21 expression may protect fibroblasts, among other cells from cell death, again underlining an interplay between budding and stroma [12]. In fact, several reports now also suggest the correlation between the presence of particular myxoid/immature stroma and the presence of tumor budding [13, 14]. We therefore hypothesize that the tumor stroma plays an important role in the tumor budding phenotype. Studies investigating the interaction between tumor buds and the surrounding microenvironment as well as the composition of a budding-promoting stroma are warranted.

A category BD0 contributes additional information on tumor behavior. Our results support previous findings, where statistical models are used to determine the probability of tumor budding counts with lymph node and distant metastasis as endpoints, underlining the additive effect of single tumor budding counts in a continuous manner [15]. A tumor with BD0 is significantly more likely to be pT1 compared to BD1 cancers (46.3% versus 16.6%) and consequently are of earlier TNM stage. Conversely, of the 139 pT1 tumors in our series, 62% are BD0. A complete absence of tumor budding is also more frequently found in well-differentiated cancers and have less vessel invasion.

Despite the large sample size of the original cohort (*n* = 959), a subgroup analysis of pT1 cancers results in low numbers of cases (*n* = 86), of which only 9 present with lymph node metastasis. The contribution of BD0 or a new 4-tier system for budding scores with relation to clinical impact in pT1 cases cannot be confirmed. Our preliminary results showing a high number of BD0 cases in pT1 tumors warrants further analysis in a larger pT1 cohort. In addition, overall and disease-free survival information was available for more than 350 patients in this study, but again only 37 patients have a BD0 score, which limits us from drawing conclusions related to patient outcome. Follow-up information for the remaining patients is still short.

On the other hand, the sample size and mixed cohort allows us to study associations of BD0 with histopathological features. It is a retrospective and prospective collection of ITBCC scores and data obtained from a real-life diagnostic setting with numerous reporting pathologists. To conclude, the results of the study suggest that the addition of a “BD0” category to the ITBCC scores has value. It occurs relatively frequently, and is indicative of significantly less aggressive colorectal cancer compared to those with any degree of tumor budding. BD0 should be considered for inclusion in subsequent ITBCC guidelines.
